# Role of empirical isolation of the superior vena cava in patients with recurrence of atrial fibrillation after pulmonary vein isolation—a multi-center analysis

**DOI:** 10.1007/s10840-022-01314-w

**Published:** 2022-08-18

**Authors:** Sven Knecht, Ivan Zeljkovic, Patrick Badertscher, Philipp Krisai, Florian Spies, Jan Vognstrup, Nikola Pavlovic, Sime Manola, Stefan Osswald, Michael Kühne, Christian Sticherling

**Affiliations:** 1 Department of Cardiology, University Hospital Basel, Petersgraben 4, 4031 Basel, Switzerland; 2 Cardiovascular Research Institute Basel, University Hospital Basel, Basel, Switzerland; 3 Department of Cardiology, Sestre Milosrdnice University Hospital, Zagreb, Croatia; 4 Department of Cardiology, Dubrava University Hospital, Zagreb, Croatia

**Keywords:** Superior vena cava, Isolation, Repeat, Pulmonary vein isolation, Atrial fibrillation, Recurrence

## Abstract

**Background:**

Non-pulmonary vein (PV) triggers play a role in the initiation of atrial fibrillation (AF), with the superior vena cava (SVC) being a common location. The aim of the current study was to investigate a strategy of empirical SVC isolation (SVCI) in addition to re-isolation of PV in patients with recurrence of AF after index PV isolation (PVI).

**Methods:**

We retrospectively analyzed consecutive patients from two centers with recurrence of AF after index PVI, undergoing a repeat ablation. Whereas only a re-isolation of the PV was intended in patients with reconnections of equal or more than two PV (PVI group), an additional SVCI was aimed for in patients with < 2 isolated PV in addition to the re-isolation of the PV (PVI + group). Analysis was performed as-treated and per-protocol.

**Results:**

Of the 344 patients included in the study (age 60 ± 10 years, 73% male, 66% paroxysmal AF), PVI only was performed in 269 patients (77%) and PVI plus SVCI (PVI +) in 75 patients (23%). Overall, freedom from AF/AT after repeat PVI was 80% (196 patients) in the PVI group and 73% in the PVI + group (*p* = 0.151). In multivariable Cox regression analysis, presence of persistent AF (HR 2.067 (95% CI 1.389–3.078), *p* < 0.001) and hypertension (HR 1.905 (95% CI 1.218–2.980), *p* = 0.005) were identified as only significant predictors of AF/AT recurrence. The per-protocol results did not differ from this observation.

**Conclusions:**

A strategy of an empirical additional SVCI at repeat PVI ablation for recurrence of AF/AT does not improve outcome compared to a PVI only approach.

**Supplementary Information:**

The online version contains supplementary material available at 10.1007/s10840-022-01314-w.

## Introduction

Atrial fibrillation (AF) is initiated by pulmonary vein or atrial triggers and perpetuated by the atrial substrate. Triggers outside of the pulmonary veins (PV) are mainly identified at the inferior mitral annulus, left atrium (LA) posterior wall, the area around the fossa ovalis region of the interatrial septum (IAS), the crista terminalis, and the coronary sinus as well as the superior vena cava (SVC) [1]. Furthermore, the left atrial appendage (LAA) [2] and the ligament of Marshall [3] have been identified as regions showing triggered activity. These anatomically defined regions are supposed to be mainly responsible for AF induction in patients with paroxysmal AF, but triggers that sustain AF were seen in at least 50% of patients independent of the type of AF [1]. In addition, LA triggers arise from low-voltage areas in the LA corresponding to fibrotic tissue [4]. Out of all these trigger locations, the SVC with atrial muscle sleeves similar to the PV [5] has been identified as one of the common non-PV foci (NPVF). Consequently, it is considered to be a relevant and anatomical amenable target for the treatment of AF. The prevalence of ectopy from the SVC is thought to be in the range of 20%, which is similar to triggers arising from the non-PV LA triggers [6, 7]. A limited number of small randomized studies on empirical SVC isolation (SVCI) in addition to the PV isolation (PVI) at index ablation showed inconclusive results with a trend for reduction of AF recurrence for patients with paroxysmal AF only [8–10].

In repeat procedures in patients with recurrence of AF after PVI, the number of reconnected veins was associated with a lower recurrence rate during follow-up [11]. Furthermore, an empirical compared to a signal-based SVCI strategy in addition to PVI was shown to be superior in repeat procedures [12]. However, the difference of a PVI only versus PVI and SVCI ablation strategy after index PVI has not yet been investigated.

The aim of our study was to assess the role of an empirical strategy of SVCI in addition to PV re-isolation during repeat procedure in patients with recurrence of AF after index PVI using a multicenter dataset.

## Methods

### Study population

We performed a dual-center, non-randomized, registry-based study. Consecutive patients with documented recurrence of AF with a duration of > 30 s after index PVI, who underwent a repeat ablation in two centers between 2010 and 2017, were included. Index ablation was performed using a cryoballoon (CB) catheter or an irrigated radiofrequency (RF) ablation catheter in combination with a 3D electroanatomical mapping (EAM) system with confirmed entrance block into the vein as procedural endpoint. Exclusion criteria were patients with long-standing AF and lesions in addition to PVI performed during index and repeat ablation. All patients gave written informed consent prior to the procedure. The study was approved by the local ethics committee and performed in accordance with the Declaration of Helsinki. Data are available on request.

### Ablation strategy

Repeat ablation was performed using RF energy with an irrigated tip catheter (Navistar Thermocool, ST & SF, Biosense Webster, Diamond Bar, CA, USA) in combination with a 3D EAM system (Carto3, Biosense Webster). After transseptal puncture under fluoroscopic guidance, the ablation catheter and a variable circular mapping catheter (Lasso, Biosense Webster) were advanced to the LA. A fast anatomical mapping (FAM) was performed using the circular mapping catheter with a resolution of 20 and respiratory compensation. Pulmonary vein reconnection was assessed based on local PV signals defined by the circular mapping catheter at the anatomical ostium on the FAM reconstruction of the LA [13]. The PV was considered to be isolated if no local PV signals were recorded (entrance block). After LA mapping, the ablation strategies were as follows: With reconnections of equal or more (≥ 2) than two PV, isolation of the PV only, without any additional lesions (PVI group), was intended. With fewer than two reconnected PV (≤ 1), SVCI in addition to the re-isolation of the PV (PVI + group) was intended. The final decision and implementation, however, was at the discretion of the physician. To perform PV re-isolation, RF energy was delivered with 25 watts (W) at the posterior wall of the LA and the SVC and 30 W at the anterior wall. After PVI, the variable circular mapping catheter was retracted to the RA and a detailed FAM of the SVC and its junction to the RA was performed. Before ablation at the ostium of the SVC, the variable circular mapping catheter was placed 1 cm in the SVC. Pacing was performed using the ablation catheter with an output of 12 V and 2.9 ms to exclude local phrenic nerve capture at this location. In case of phrenic nerve capture, the ablation was performed at 20 W or omitted. If an ablation was performed, capture of the phrenic nerve was confirmed after ablation. The endpoint of the ablation was the elimination of the local SVC signals after a circumferential lesion set (entrance block). Antiarrhythmic drug therapy was stopped after ablation in patients with paroxysmal AF. For patients with persistent AF, it was continued for the blanking period of three months at the discretion of the treating physician.

### Follow-up and outcome analysis

After a blanking period of 3 months, follow-up visits including a detailed history and physical examination were performed at 3 and 6 months after the procedure with a 24-h Holter-ECG and a 7-day Holter-ECG monitoring at 12 months. In patients with symptomatic recurrences, 12-lead ECG and 24-h Holter monitoring were performed to document the tachycardia. Episodes of AF or any sustained left atrial tachycardia (AT) (> 30 s) were counted as recurrences. Complications were defined as major when resulting in prolongation of the hospital stay, requiring additional intervention or resulting in significant injury or death.

### Statistical analysis

Continuous variables are presented as mean ± one standard deviation and median with interquartile ranges (IQR). For continuous variables, comparisons were made using Student’s *T*-test, or Mann–Whitney *U* test, as appropriate. Test for normality was performed using the Kolmogorov–Smirnov test. Discrete variables were compared using Fisher’s exact test. Kaplan–Meier survival curve analysis with a log-rank test was used to determine the probability and test the difference of the freedom from AF recurrence for the two groups. To account for non-adherence to the treatment advice based on the number of reconnected veins, we performed a per-protocol analysis in addition to the as-treated analysis, excluding patients that were not treated according to the treatment strategy.

A univariable and multivariable Cox regression model using a stepwise forward approach was performed to identify predictors of recurrence of AF/AT for both analyzing methods. All analyses were performed using SPSS (version 22.0, SPSS Inc., Chicago, IL) and a *p*-value < 0.05 was considered statistically significant.

## Results

### Study population

Out of a total of 1359 patients with PVI only at index ablation, 548 patients showed AF recurrence. Thereof, a repeat procedure was performed in 391 patients. Of these patients with a repeat procedure, additional ablations were reformed in 15 patients (mainly CFAE, roof line, mitral isthmus line, box lesion, due to low voltage areas). Of the remaining 376 patients, 32 were lost to follow-up, resulting in 344 patients included in the study (Supplemental Fig. 1). PVI only (PVI group) was performed in 269 patients (77%) and PVI plus SVCI (PVI + group) in 75 patients (23%) (as-treated). Mean age of the patients was 60 ± 10 years, 73% were men, and 228 patients (66%) had paroxysmal AF (Table 1). Median follow-up after the repeat PVI was 320 days with no significant difference between the two groups (PVI 265 ± 113 days (median 313 days), PVI + 273 ± 112 days (median 350 days), *p* = 0.822). Index ablation was performed using RF or CB ablation in 260 (76%) and 84 (24%) patients, respectively. No difference in AF/AT recurrence rate after repeat procedure was observed between the two modalities used for index ablation (RF energy 28%, CB 32%; *p* = 0.488). Baseline characteristics for the per-protocol analysis, which show comparable baseline data to the as-treated analysis, are summarized in the supplemental Table 1. Per-protocol analysis resulted in a total of 272 patients. Thereof, a repeat PVI was performed in 235 patients (86%) and an additional SVCI was performed in 37 patients (14%).

**Fig. 1:**
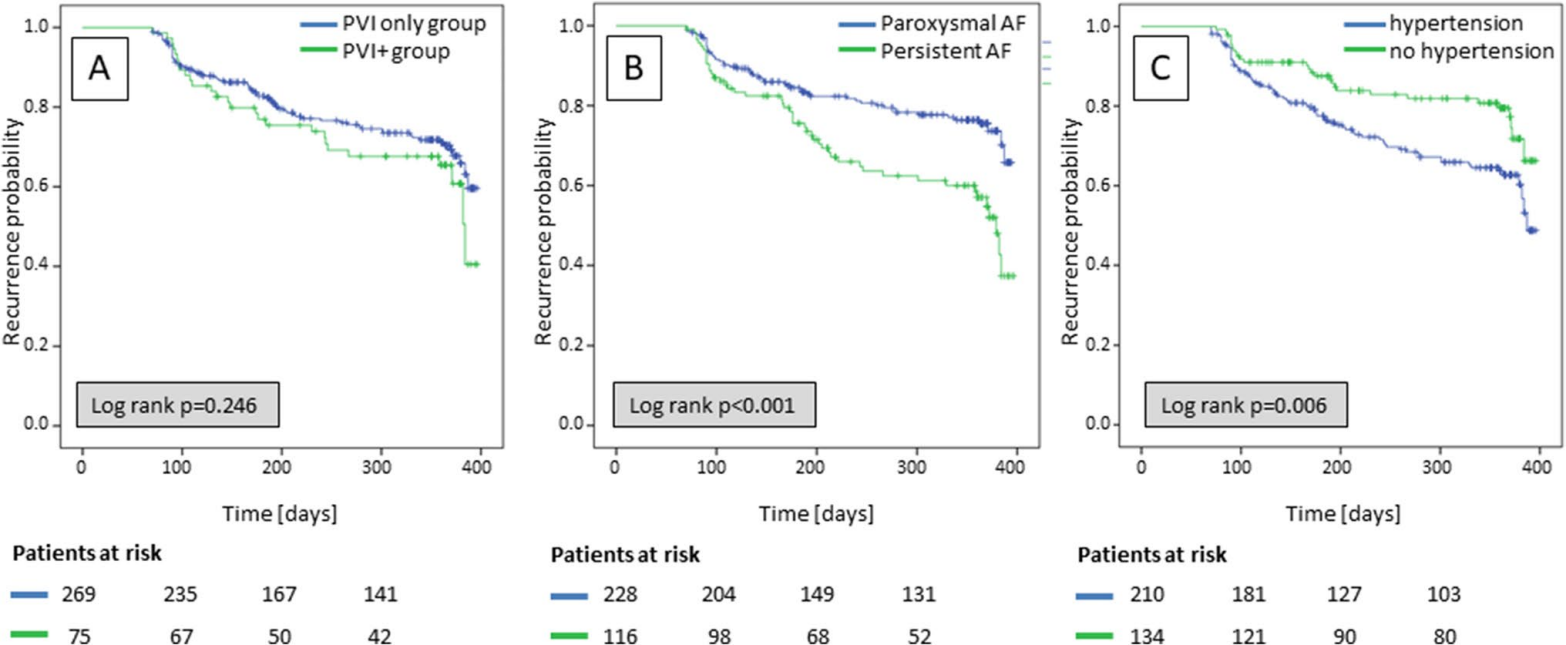
Kaplan–Meier survival curves stratified by (**A**) the ablation group, (**B**) the prevalence of paroxysmal AF, and (**C**) the prevalence of hypertension

**Table 1:** Baseline data: AS-TREATED

Parameter	All (*n* = 344)	PVI group *n* = 269	PVI + group *n* = 75	*P*-value
Demographics				
Male sex	251 (73%)	195 (72%)	56 (75%)	0.770
Age (years)	60 ± 10 (61)	60 ± 10 (61)	61 ± 9 (62)	0.421
BMI (kg/m^2^)	28 ± 4 (27)	28 ± 4 (27)	26 ± 4 (27)	0.006
Paroxysmal AF	228 (66%)	180 (67%)	48 (64%)	0.679
Duration of AF (month)	55 ± 60 (40)	51 ± 62 (40)	60 ± 57 (43)	0.079
PLAX (mm)	41 ± 6 (41)	41 ± 7 (41)	41 ± 5 (41)	0.618
LVEF (%)	58 ± 8 (60)	59 ± 9 (60)	58 ± 7 (60)	0.917
LAVI (ml/m^2^)	36 ± 12 (34)	35 ± 12 (33)	37 ± 11 (37)	0.073
Comorbidities				
CAD	14 (4%)	11 (4%)	3 (4%)	0.755
Smoking				0.414
Yes	41 (12%)	30 (11%)	11 (15%)	
No	179 (52%)	137 (51%)	42 (56%)	
Past	120 (35%)	98 (36%)	22 (29%)	
HT	210 (61%)	168 (62%)	42 (56%)	0.349
Diabetes	25 (7%)	21 (8%)	4 (5%)	0.572
Renal insufficiency	21 (6%)	18 (7%)	3 (4%)	0.515
CHADSVASC				0.276
0	79 (23%)	62 (23%)	17 (23%)	
1	103 (30%)	81 (30%)	22 (29%)	
2	78 (23%)	56 (21%)	22 (29%)	
3	50 (15%)	42 (16%)	8 (11%)	
4	21 (6%)	19 (7%)	2 (3%)	
≥ 5	13 (4%)I	9 (3%)	4 (5%)	
Procedural parameters				
RF-PVI at index	244 (76%)	187 (78%)	73 (73%)	0.491
Procedure duration (min)	108±40 (104)	110 ± 42 (105)	98 ± 34 (97)	0.310
Reconnected veins	2.4 ± 1.1 (2)	2.6 ± 1.0 (3)	1.5 ± 1.0 (2)	< 0.001
0	15 (4%)	2 (1%)	13 (17%)	
1	56 (16%)	32 (12%)	24 (32%)	
2	124 (36%)	94 (35%)	30 (40%)	
3	89 (26%)	83 (31%)	6 (9%)	
4	60 (17%)	58 (22%)	2 (2%)	
Location				
LSPV (341)	187 (55%)	164 (62%)	23 (30%)	< 0.001
LIPV	170 (49%)	153 (58%)	17 (22%)	< 0.001
RSPV	207 (60%)	173 (65%)	34 (44%)	0.002
RIPV	237 (69%)	198 (75%)	39 (51%)	< 0.001
RF duration				
Overall	849±482 (769)	859 ± 491(778)	779 ± 416 (720)	0.244
PVI	791± 494 (695)	859 ± 491 (778)	511 ± 344 (468)	< 0.001
SVC	57±142 (0)	0	265 ± 196 (211)	< 0.001

Values are *n* (%) for categorical and mean ± standard deviation (median) for continuous variables

*AF*, atrial fibrillation; *AFB*, AF burden score; *BMI*, body mass index; *CHADSVASC*, (*n* = 343); *PLAX*, parasternal long-axis (*n* = 284); *LAVI*, left atrial volume indexed (*n* = 249); *LVEF*, left ventricular ejection fraction (*n* = 333)

Duration of AF (*n* = 337) was defined as the time interval between the first diagnosis of AF and pulmonary vein isolation

### Procedural differences

Reconnection of 0, 1, 2, 3, and 4 PV were observed in 15 (4%), 56 (16%), 124 (36%), 89 (26%), and 60 patients (17%), respectively. The right inferior pulmonary vein (RIPV) was the most frequently reconnected vein in both study groups (Table1). The number of reconnected PV was lower in the PVI group compared to the PVI + group both according to the as-treated and the per-protocol analysis (< 0.001). Complete isolation of the SVC was possible in 72 patients (96%), whereas SVCI could not be performed in three patients due to phrenic nerve capture at the targeted ablation site. These patients remained in the PVI + group. No transient or persistent phrenic nerve palsy (PNP) was observed in the study groups.

The mean procedure time was 107 ± 40 min (110 ± 42 min in PVI group vs. 99 ± 34 min in PVI + group; *p* = 0.063). The mean fluoroscopy time was 4 ± 7 min with a significant difference between the groups (5 ± 7 min in PVI only vs. 3 ± 6 min in PVI + ; *p* = 0.048). RF duration for achieving PVI but not overall RF duration differed between the PVI only and PVI + group (859 ± 491 and 511 ± 344 s; *p* < 0.001; and 859 ± 491 and 779 ± 416 s, *p* = 0.244; respectively). Procedural data of the per-protocol analysis are summarized in the supplemental Table 1, showing results comparable to the as-treated analysis.

### Recurrent atrial fibrillation after repeat ablation

Freedom from AF/AT after the repeat PVI was 80% (196 patients) in the PVI only and 73% in the PVI + group (*p* = 0.151). Compared to patients with AF/AT recurrence, patients without AF/AT recurrence had a higher prevalence of paroxysmal AF (72 vs. 53%, *p* < 0.001), a lower LA size (41 ± 7 vs. 43 ± 6 mm, *p* = 0.033), lower indexed LA volume (35 ± 12 vs. 38 ± 11 ml/m^2^, *p* = 0.009), lower prevalence of hypertension (57 vs. 72%, *p* = 0.005), and a higher CHA_2_DS_2_VASc score (*p* = 0.05). No differences were found between the two groups concerning the additional isolation of the SVC, the number of reconnected veins, or the cumulative RF time for PVI (Table 2). No major complications were observed in either of the two study groups.

**Table 2:** One-year freedom from AF: AS-TREATED

Parameter (*n* = 344)	No recurrence *n* = 244	Recurrence *n* = 100	*P*-value
Male sex	184 (75%)	67 (67%)	0.073
Age	60 ± 10 (61)	61 ± 10 (62)	0.270
BMI	28 ± 4 (27)	26 ± 4 (27)	0.882
Paroxysmal AF	176 (72%)	52 (53%)	< 0.001
Duration of AF (month)	55 ± 60	69 ± 62	0.392
PLAX (mm)	41 ± 7 (41)	43 ± 6 (42)	0.033
LVEF (%)	58 ± 9 (60)	59 ± 7 (60)	0.130
LAVI (ml/m^2^)	35 ± 12 (33)	38 ± 11 (37)	0.009
Comorbidities			
CAD	11 (5%)	3 (3%)	0.534
Smoking (340)			0.334
Yes	26 (11%)	15 (15%)	0.516
No	126 (51%)	53 (53%)	
Past	88 (36%)	32 (32%)	
HT	138 (57%)	72 (72%)	0.005
Diabetes	16 (7%)	9 (9%)	0.547
Renal insufficiency	12 (5%)	9 (9%)	0.242
CHADSVASC			0.050
0	64 (26%)	15 (15%)	
1	70 (29%)	33 (33%)	
2	57 (23%)	21 (21%)	
3	31 (13%)	19 (19%)	
4	17 (7%)	4 (4%)	
≥ 5	6 (2%)	7 (7%)	
Procedural			
Procedure duration (min)	106 ± 39 (102)	112 ± 43 (109)	0.152
Fluoroscopy duration (min)	9 ± 7 (8)	9 ± 8 (7)	0.702
RF-PVI at index	181 (74%)	73 (73%)	0.491
SVCI	48 (20%)	27(27%)	0.151
Number of reconnected veins	2.6 ± 1.0 (3)	1.5 ± 1.0 (2)	0.508
			0.853
0	9 (4%)	6 (6%)	
1	38 (16%)	18 (18%)	
2	90 (37%)	34 (34%)	
3	64 (26%)	25 (25%)	
4	43 (18%)	17 (17%)	
Location			
LSPV (341)	134 (55%)	53 (53%)	0.904
LIPV	123 (50%)	47 (47%)	0.720
RSPV	152 (62%)	55 (55%)	0.226
RIPV	169 (69%)	68 (68%)	1.000
PVI RF duration	790 ± 482 (713)	795 ± 524 (673)	0.804
SVC duration	49 ± 127 (0)	78 ± 171 (0)	0.096

Values are *n* (%) for categorical and mean ± standard deviation and median (median) for continuous variables

Duration of AF (*n* = 337) was defined as the time interval between the first diagnosis of AF and pulmonary vein isolation

*AF*, atrial fibrillation; *AFB*, AF burden score; *BMI*, body mass index; *CHADSVASC*, (*n* = 343); *PLAX*, parasternal long-axis (*n* = 284); *LAVI*, left atrial volume indexed (*n* = 249); *LVEF*, left ventricular ejection fraction (*n* = 333)

The Kaplan–Meier survival analysis showed no difference in AF/AT recurrence between the two groups, neither for the complete study cohort (*p* = 0.246) nor for a sub-analysis of the patients with paroxysmal AF (*p* = 0.154). However, differences were observed for prevalence of paroxysmal AF and hypertension (Fig. 1).

Results from the per-protocol analysis showed comparable results. In detail, freedom from AF/AT after the repeat PVI was 74% (173 patients) in the PVI only and 65% (24 patients) in the PVI + group (*p* = 0.322). Compared to patients with AF/AT recurrence, patients without AF/AT recurrence had a higher prevalence of paroxysmal AF (69 vs. 62%, *p* < 0.030) and a lower prevalence of hypertension (57 vs. 72%, *p* = 0.012) (Supplemental Table 2). Kaplan–Meier survival analysis showed no difference in AF/AT recurrence between the two study groups (*p* = 0.417).

### Prediction of AF/AT recurrence

In univariable Cox regression analysis, paroxysmal AF, indexed LA volume, LVEF, prevalence of hypertension, and the CHA_2_DS_2_VASc score were identified as significant predictors of AF/AT recurrence after the repeat procedure (Table 3). In multivariable Cox regression analysis corrected for BMI due its difference in baseline data, presence of persistent AF and hypertension were identified as only significant predictors of AF/AT recurrence (HR (95% CI) 2.067 (1.398–3.078), *p* < 0.001; HT HR (95% CI) 1.905 (1.218–2.980), *p* = 0.005). None of the procedural parameters, including SVCI and the number of reconnected PV predicted AF/AT recurrence (Table 3). Results for the cox regression of the per-protocol analysis, which also show the presence of persistent AF and hypertension as only significant predictors of AF/AT recurrence, are summarized in supplemental Table 3.

**Table 3:** Regression analysis of predictors of arrhythmia freedom: AS-TREATED

Parameter	Univariate HR (95% CI)	*P* value	Multivariate HR (95% CI)	*P* value
Male sex	0.702 (0.462–1.065)	0.096		
Age	1.012 (0.991–1.033)	0.255		
BMI	1.017 (0.970–1.065)	0.483	0.989 (0.943–1.037)	0.648
Persistent AF	2.002 (1.352–2.965)	< 0.001	2.067 (1.398–3.078)	< 0.001
Duration of AF (month) (*n* = 338)	1.001 (0.998–1.004)	0.664		
PLAX (mm)	1.030 (0.999–1.061)	0.056		
LVEF (%)	0.979 (0.959–1.000)	0.048		
LAVI (ml/m^2^)	1.020 (1.003–1.038)	0.022		
Comorbidities				
CAD	0.753 (0.239–2.376)	0.628		
Smoking				
Past (reference)				
Yes	1.264 (0.874–1.828)	0.213		
No	0.975 (0.742–1.281)	0.853		
HT	1.824 (1.179–2.828)	0.007	1.905 (1.218–2.980)	0.005
Diabetes	0.787 (0.446–1.389)	0.408		
Renal insufficiency	0.854 (0.468–1.558)	0.607		
CHADSVASC	1.206 (1.054–1.386)	0.009		
0				
1				
2				
3				
4				
≥ 5				
Procedural				
Procedure duration (min)	1.003 (0.999–1.008)	0.158		
Fluoroscopy duration (min)	1.000 (0.974–1.027)	0.991		
RF-PVI at index	0.850 (0.546–1.322)	0.470		
SVCI	1.300 (0.836–2.022)	0.244		
Number reconnected veins	0.937 (0.779–1.128)	0.937		
PV RF duration	1.000 (1.000–1.000)	0.795		
SVC duration	1.001 (1.000–1.002)	0.079		

*BMI*, body mass index; *CAD*, coronary artery disease; *HT*, hypertension; *PLAX*, parasternal long-axis (*n* = 283); *LVEF*, left ventricular ejection fraction (*n* = 332); *LAVI*, left atrial volume indexed (*n* = 248)

## Discussion

The purpose of this study was to investigate whether an empirical strategy of SVCI during repeat PVI in patients with recurrence of AF after an index PVI improves the outcome as compared to no additional lesions to PVI. The main observations of our study are as follows: (1) Patients who received empirical SVCI in addition to re-isolation of the PV did not differ in AF/AT recurrence rate compared to patients with PVI only during repeat procedure. A benefit of SVCI was not observed, neither when selectively analyzing patients with < 2 reconnected veins nor with ≥ 2 reconnected PV. (2) No difference between the groups was found even when analyzing only patients with paroxysmal AF. (3) The established risk factors (LA size from PLAX, indexed LA volume, hypertension, and AF type) differed between the two groups with and without AF/AT recurrence. Thereby, the prevalence of hypertension and persistent AF were identified as significant predictors of AF/AT recurrence after the repeat PVI procedure. (4) The above-described observations hold true both, for the as treated and the per-protocol analysis.

NPVF in patients with AF were identified in both atrial chambers, with posterior wall being the most common in LA and SVC in the right atrium, but as well the most common overall [1–3, 8–10]. The prevalence of ectopy from the SVC is estimated to be up to 20% [6, 7]. However, studies on catheter ablation for the treatment of AF that focus on PVI alone compared to a combination of PVI and SVCI at index ablation are scarce and show inconsistent results [8–10]. Out of the three randomized controlled trials using RF energy [14], only one study demonstrated an improved outcome of empirical SVCI at index ablation and this was only the case in patients with paroxysmal AF [10]. Of note, compared to as-needed SVCI, empirical SVCI, as performed in our study, has been shown to results in lower AF/AT recurrence after index ablation [12]. Recently, a retrospective study by a highly experienced CB center demonstrated both the feasibility as well as a lower AF/AT recurrence when performing SCVI using the CB in patients with paroxysmal AF. However, the incidence of PNP and sinus node impairment was increased [15].

For repeat procedures in patients with AF/AT recurrence after index PVI, there currently only exists one study which investigates the value of additional SVCI on top of PVI [12]. It is suspected that the likelihood of NPVF as a potential trigger to initiate AF increases after repeat PVI due to the higher probability of permanently isolated PV. This assumption would help to investigate the real value of the SVCI in addition to PVI and it would be less confounded by AF recurrence due to reconnected veins. However, technology development in PVI, especially balloon-based technologies, allow for a quick and easy PVI with a lower number of reconnected veins detected at repeat procedures. This effect is more pronounced for CB compared to RF PVI [16]. In consequence, additional ablation strategies for patients with AF recurrence after index PVI despite no or a low number of reconnected PV, are required. In a recent study, Inamura et al. analyzed the location of NPVF identified in 564 of 2967 screened patients after PVI. After the exclusion of PV triggers, the SVC was identified as the location of NPVF in 38% (213 patients) of these patients [17]. By means of multivariate analysis, they identified female gender, low BMI, and non-paroxysmal AF as predictors for NPVF from the IAS, the coronary sinus, and the RA in general. The low BMI as predictor for ectopic firing form the SVC was confirmed in another study [18]. Of note, no predictors for the SVC as NPVF were identified, despite its high prevalence. Since, in our study, the patients with AF recurrence also had non-paroxysmal AF more often and showed a trend towards being more women (33% with vs. 25% without recurrence, *p* = 0.073), the AF recurrence in our cohort might be partially explained by triggers outside the PV and the SVC. Another study comparing index with repeat ablation reported the incidence of SVC triggers to be dominant during index ablation (52% of all NPVF were identified in the SVC after exclusion of PV as triggers), but not during repeat procedure (35% of all NPVF) [19]. This was mainly due to the fact that the 82 new-identified NPVF at repeat ablation were more broadly distributed over the different locations, which decreased the overall percentage of SVC triggers. The possible reasons for the difference of NPVF before and after index ablation are diverse: (1) the modulation of the autonomic tone during index ablation might have impaired the suppression of NPVF. (2) LA fibrosis due to disease progression and ablation itself might result in more NPVF. (3) The septum puncture during the index procedure might explain the higher number of IAS foci during repeat procedures (13% at index vs. 22% during repeat ablation). The latter potential reason for NPVF was observed in another study on repeat ablation with a relatively high prevalence of IAS foci of 18% [20]. These differences in the prevalence of NPVF between first and repeat ablation might explain our results, which, in contrast to previous studies at index ablation, showed no differences between the two groups even for patients with paroxysmal AF [10, 15]. Of note, in accordance with a previous study with patients with paroxysmal AF [21], we observed no impact of the index ablation modality (CB vs. focal RF PVI) on the outcome in our study.

Although our data does not clarify the percentage of NPVF stemming from the SVC, it clearly shows that there is no clinical benefit from empirical ablation.

## Limitations

Despite the combination of patients from two centers, this study still included a relatively small number of patients with SVCI. Since it is not a randomized study, differences between the groups were observed for baseline data. We addressed this potential confounder by correcting for BMI in the multivariable Cox regression analysis. The ablation strategy (additional SVCI in patients with ≤ 1 reconnected PV; PVI with equal or more than 2 reconnected PV) was not implemented in all of the patients, since the decision concerning the strategy was ultimately at the discretion of the physician. However, the results from a per-protocol did not differ from the results of the as-treated analysis. We assessed a strategy of an empirical SVCI in addition to the PV re-isolation based on the number of reconnected vein. Conclusions on the value of SVCI independent on the number of reconnected PV cannot be drawn.

A potential bias exists due to the exclusion of patients with lesions in addition to PVI, SCVI, or CTI, such as mitral isthmus line or roof line in patients with documented left atrial flutter, or large low voltage areas in the LA. Whereas AAD was stopped after ablation in one center, AAD could be continued for a maximum of three months at the discretion of the physician in the other center. This could in theory results in later recurrence of AF in some patients. Furthermore, we did not assess the length of the SVC sleeves or the potential, which was identified as predictor of a SVC focus, due to the purely empirical strategy [22]. Finally, reconnection of the SVC sleeves in addition to the reconnection of PV sleeves might be a reason for AF recurrence.

## Conclusion

A strategy of additional SVCI at repeat PVI ablation for recurrence of AF/AT does not improve outcome compared to a PVI only approach. This holds true for the as-treated and per-protocol analysis as well for patients with paroxysmal AF. The only independent predictors of recurrence after repeat PVI were the presence of hypertension and persistent AF type.

## Supplementary Information

Below is the link to the electronic supplementary material.

ESM 1 (PNG 623 kb)

High Resolution Image (TIF 102 kb)

Supplementary file2 (DOCX 24 KB)
