# Supplementary figures and images for: Role of empirical isolation of the superior vena cava in patients with recurrence of atrial fibrillation after pulmonary vein isolation—a multi-center analysis
Source: J Interv Card Electrophysiol. 2022 Aug 18;66(2):435–43. doi: 10.1007/s10840-022-01314-w (PMC9977848; doi:10.1007/s10840-022-01314-w)

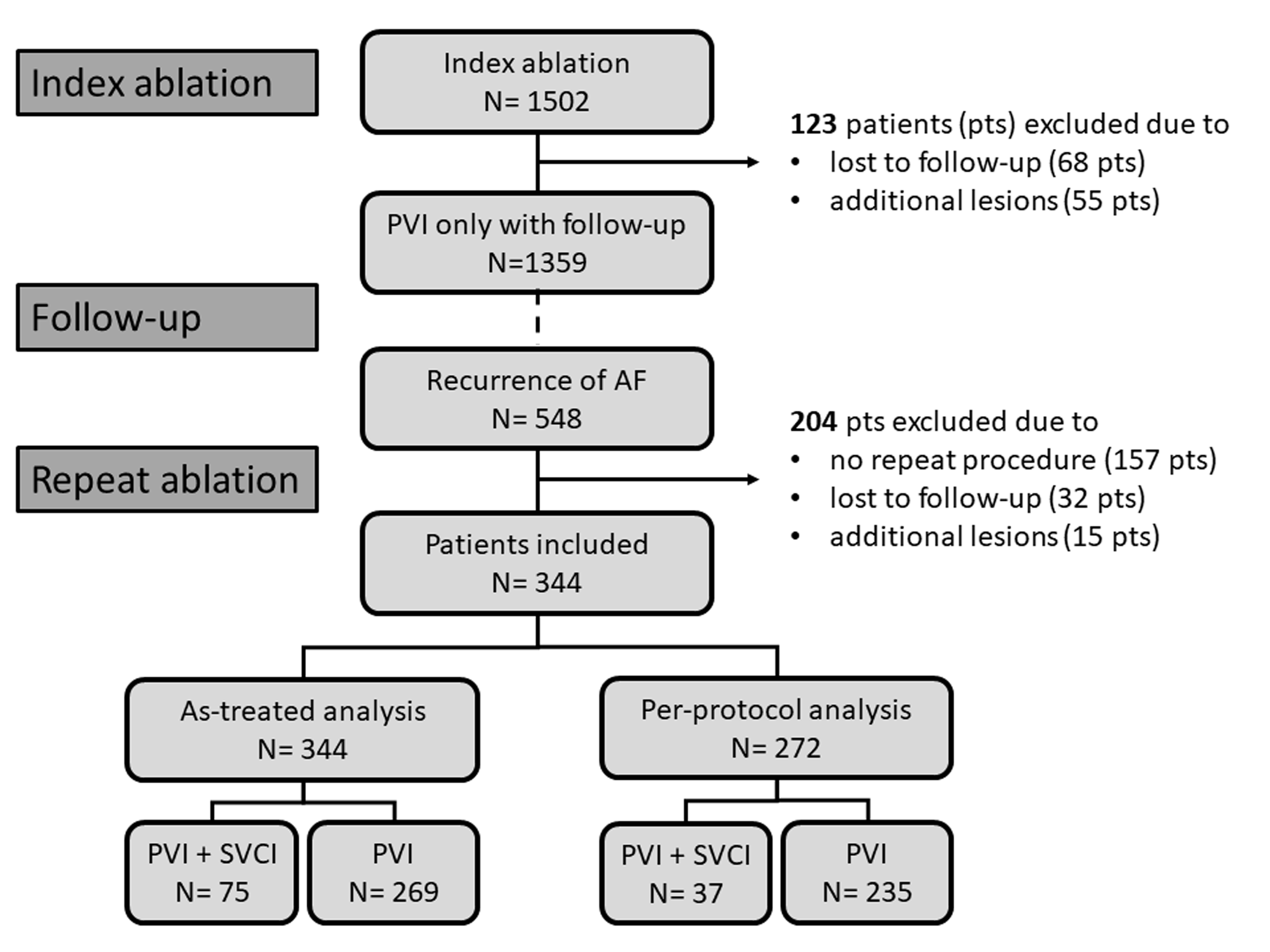

Supplement: Supplementary file 1 — (PNG 623 kb) [file 10840_2022_1314_Fig2_ESM.png]

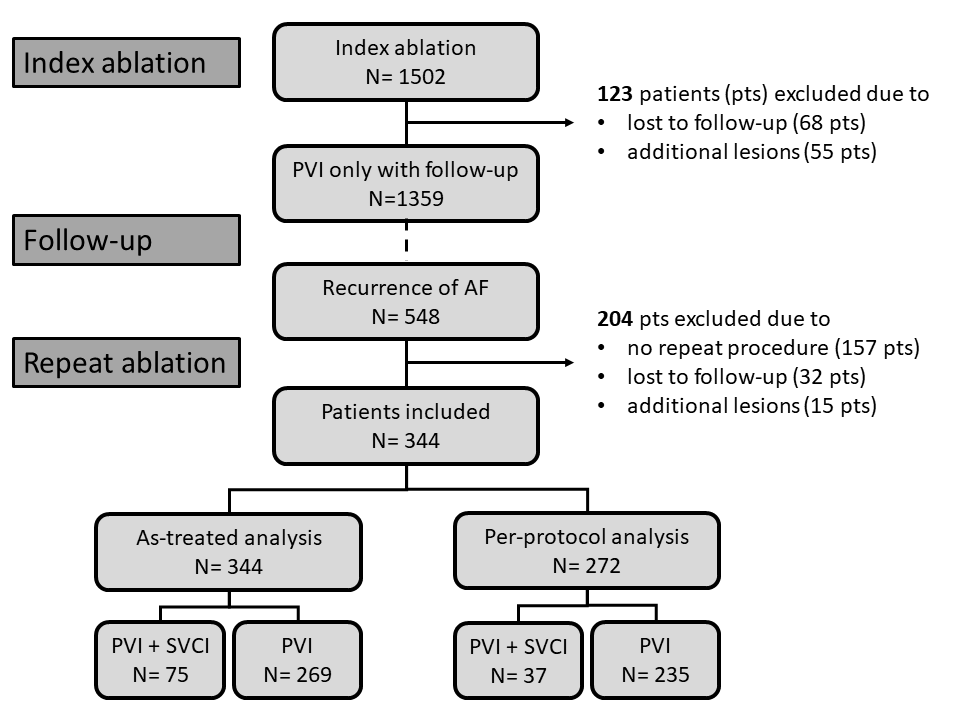

Supplement: Supplementary file 2 — High Resolution Image (TIF 102 kb) [file 10840_2022_1314_MOESM1_ESM.tif]
