# Supplementary material for: Role of empirical isolation of the superior vena cava in patients with recurrence of atrial fibrillation after pulmonary vein isolation—a multi-center analysis
Source: J Interv Card Electrophysiol. 2022 Aug 18;66(2):435–43. doi: 10.1007/s10840-022-01314-w (PMC9977848; doi:10.1007/s10840-022-01314-w)
Supplement: Supplementary file 3 — Supplementary file2 (DOCX 24 KB) [file 10840_2022_1314_MOESM2_ESM.docx]

**Supplemental Material**

**Figures**

Supplemental Figure 1: Flowchart of study procedure and analysis

**Tables**

Supplemental Table 1: Baseline Data: PER-PROTOCOL

| Parameter | All (n=272) | PVI group  N=235 | PVI+ group  N=37 | P-Value |
| --- | --- | --- | --- | --- |
| **Demographics** |  |  |  |  |
| Male sex | 199 (73%) | 173 (72%) | 26 (75%) | 0.692 |
| Age [years] | 60±10 (61) | 59±10 (60) | 61±9 (63) | 0.344 |
| BMI [kg/m^2^] | 28±4 (27) | 28±4 (27) | 26±3 (26) | 0.039 |
| Paroxysmal AF | 184 (68%) | 161 (69%) | 23 (62%) | 0.454 |
| Duration of AF [months] | 59±58 (42) | 57±57 (41) | 70±66 (45) | 0.372 |
| **Comorbidities** |  |  |  |  |
| PLAX [mm] | 41±6 (41) | 41±7 (41) | 40±5 (39) | 0.252 |
| LVEF [%] | 59±8 (59) | 59±8 (60) | 60±7 (60) | 0.510 |
| LAVI [ml/m^2^] | 34±11 (33) | 34±10 (32) | 38±12 (35) | 0.048 |
| **Comorbidities** |  |  |  |  |
| CAD | 12 (4%) | 11 (4%) | 1 (4%) | 1.000 |
| Smoking |  |  |  | 0.629 |
| yes | 28 (11%) | 25 (11%) | 3 (8%) |  |
| no | 143 (53%) | 123 (52%) | 20 (54%) |  |
| past | 99 (36%) | 85 (36%) | 14 (28%) |  |
| HT | 166 (61%) | 144 (61%) | 22 (59%) | 0.857 |
| Diabetes | 18 (7%) | 17 (7%) | 1 (3%) | 0.483 |
| Renal insufficiency | 15 (6%) | 15 (6%) | 0 (0%) | 0.240 |
| CHADSVASC |  |  |  | 0.807 |
| 0 | 67 (18%) | 58 (25%) | 9 (24%) |  |
| 1 | 80 (29%) | 70 (30%) | 10 (27%) |  |
| 2 | 61 (26%) | 50 (21%) | 11 (30%) |  |
| 3 | 40 (15%) | 35 (15%) | 5 (14%) |  |
| 4 | 17 (6%) | 15 (6%) | 2 (5%) |  |
| ≥5 | 7 (3%)I | 7 (3%) | 0 (0%) |  |
| **Procedural parameters** |  |  |  |  |
| RF-PVI at index | 205 (76%) | 183 (78%) | 22 (59%) | 0.023 |
| Procedure duration [min] | 110±42 (105) | 113±43 (105) | 93±34 (89) | 0.009 |
| Reconnected veins |  | 2.9±0.8 (3) | 0.65±0.5 (1) | <0.001 |
| 0 | 13 (5%) | 0 (0%) | 13 (35%) |  |
| 1 | 24 (9%) | 0 (0%) | 24 (65%) |  |
| 2 | 94 (35%) | 94 (40%) | 0 (0%) |  |
| 3 | 83 (31%) | 83 (35%) | 0 (0%) |  |
| 4 | 58 (21%) | 58 (25%) | 0 (0%) |  |
| Location |  |  |  |  |
| LSPV | 166 (61%) | 157 (67%) | 9 (24%) | <0.001 |
| LIPV | 153 (56%) | 151 (64%) | 2 (5%) | <0.001 |
| RSPV | 175 (64%) | 169 (72%) | 6 (16%) | <0.001 |
| RIPV | 192 (71%) | 185 (79%) | 7 (19%) | <0.001 |
| RF duration |  |  |  |  |
| overall |  | 929±490(869) | 645±341 (577) | <0.001 |
| PVI |  | 929±490 (869) | 359±272 (313) | <0.001 |
| SVC |  | 0 | 268±186 (248) | 0.001 |
|  |  |  |  |  |

Values are n (%) for categorical and mean ± standard deviation (median) for continuous variables. AF, atrial fibrillation; AFB, AF burden score; BMI, body mass index; CHADSVASC - -(n=343); PLAX- parasternal long-axis (n=284), LAVI – left atrial volume indexed (n=249); LVEF – left ventricular ejection fraction (n=333). Duration of AF was defined as the time interval between the first diagnosis of AF and pulmonary vein isolation. Duration of AF (n=337) was defined as the time interval between the first diagnosis of AF and pulmonary vein isolation.

Supplemental table 2: 1-year freedom from AF: PER-PROTOCOL

| Parameter (N=272) | NoRecurrence  N=197 | Recurrence  N=75 | P-value |
| --- | --- | --- | --- |
| Male sex | 150 (74%) | 49 (70%) | 0.092 |
| Age | 59±10 (60) | 61±10 (62) | 0.186 |
| BMI | 28±4 (27) | 28±4 (28) | 0.803 |
| Paroxysmal AF | 141 (69) | 43 (62) | 0.030 |
| Duration of AF [months] | 57±60 (40) | 64±55 (50) | 0.111 |
| Typical flutter (n=317) |  |  |  |
| PLAX [mm] | 41±7 (40) | 42±5 (42) | 0.081 |
| LVEF [%] | 59±8 (60) | 58±7 (60) | 0.105 |
| LAVI [ml/m^2^] | 34±10 (32) | 36±11 (34) | 0.094 |
| **Comorbidities** |  |  |  |
| CAD | 9 (5%) | 3 (3%) | 1.000 |
| Smoking (340) |  |  | 0.334 |
| yes | 20 (11%) | 9 (8%) | 0.587 |
| no | 103 (53%) | 41 (54%) |  |
| past | 74 (36%) | 25 (38%) |  |
| HT | 111 (57%) | 55 (72%) | 0.012 |
| Diabetes | 12 (7%) | 6 (9%) | 0.589 |
| Renal insufficiency | 9 (5%) | 6 (9%) | 0.301 |
| CHADSVASC |  |  | 0.036 |
| 0 | 57 (29%) | 10 (13%) |  |
| 1 | 53 (27%) | 27 (36%) |  |
| 2 | 44 (22%) | 17 (23%) |  |
| 3 | 26 (13%) | 14 (19%) |  |
| 4 | 14 (7%) | 3 (4%) |  |
| ≥5 | 3 (2%) | 4 (5%) |  |
|  |  |  |  |
| **Procedural** |  |  |  |
| Procedure duration [min] | 109±40 (104) | 113±48 (108) | 0.616 |
| Fluoroscopy duration [min] | 10±7 (8) | 10±8 (7) | 0.552 |
| RF-PVI at index | 151 (74%) | 54 (73%) | 0.434 |
| SVCI | 24 (12%) | 13(17%) | 0.322 |
| Number reconnected veins | 2.6±1.0 (3) | 1.5±1.0 (2) | 0.970 |
|  |  |  | 0.722 |
| 0 | 8 (4%) | 5 (7%) |  |
|  |  |  |  |
| 1 | 16 (8%) | 8 (11%) |  |
| 2 | 72 (37%) | 22 (29%) |  |
| 3 | 60 (30%) | 23 (31%) |  |
| 4 | 41 (1%) | 17 (23%) |  |
| Location |  |  |  |
| LSPV (268) | 123 (63) | 43 (57) | 0.573 |
| LIPV | 111 (56) | 42 (56) | 1.000 |
| RSPV | 128 (65) | 47 (63) | 0.77 |
| RIPV | 139 (71) | 53 (70) | 1.000 |
| PVI RF duration | 845±490 (776) | 867±547 (738) | 0.985 |
| SVC duration | 32±111 (0) | 59±139 (0) | 0.217 |
|  |  |  |  |

Values are n (%) for categorical and mean ± standard deviation and median (median) for continuous variables. AF, atrial fibrillation; AFB, AF burden score; BMI, body mass index; PLAX- parasternal long-axis (n=274), LAVI – left atrial volume indexed (n=194); LVEF – left ventricular ejection fraction (n=262). Duration of AF was defined as the time interval between the first diagnosis of AF and pulmonary vein isolation.

Supplemental table 3: Regression analysis of predictors of arrhythmia freedom: - PER-PROTOCOL

| Parameter | Univariate  HR (95%CI) | P value | Multivariate  HR (95%CI) | P value |
| --- | --- | --- | --- | --- |
| Male sex | 0.591(0.331-1.052) | 0.074 |  |  |
| Age | 1.020(0.992-1.048) | 0.166 |  |  |
| BMI | 1.003(0.943-1.066) | 0.924 |  |  |
| Persistent AF | 1.874(1.078-3.256) | 0.026 | 2.069(1.174-3.649) | 0.012 |
| Duration of AF [months] | 1.002(0.998-1.007) | 0.368 |  |  |
| PLAX [mm] | 1.039(0.993-1.087) | 0.096 |  |  |
| LVEF [%] | 0.978 (0.945-1.013) | 0.212 |  |  |
| LAVI [ml/m^2^] | 1.023(0.994-1.053) | 0.125 |  |  |
| **Comorbidities** |  |  |  |  |
| CAD | 0.861(0.227-3.271) | 0.828 |  |  |
| Smoking |  |  |  |  |
| Past (reference) |  |  |  |  |
| yes | 1.402(0.582-3.496) | 0.468 |  |  |
| no | 0.975(0.742-1.281) | 0.853 |  |  |
|  |  |  |  |  |
| HT | 2.131 (1.188-3.821) | 0.011 | 2.238(1.234-4.061) | 0.008 |
| Diabetes | 1.341 (0.484-3.711) | 0.573 |  |  |
| Renal insufficiency | 0.845(0.342-2.088) | 0.715 |  |  |
| CHADSVASC | 1.205(0.987-1.472) | 0.067 |  |  |
| 0 |  |  |  |  |
| 1 |  |  |  |  |
| 2 |  |  |  |  |
| 3 |  |  |  |  |
| 4 |  |  |  |  |
| ≥5 |  |  |  |  |
| **Procedural** |  |  |  |  |
| Procedure duration [min] | 1.002(0.996-1.008) | 0.518 |  |  |
| Fluoroscopy duration [min] | 1.001(0.966-1.037) | 0.991 |  |  |
| RF-PVI at index | 0.783(0.429-1.431) | 0.427 |  |  |
| SVCI | 1.511(0.725-3.151) | 0.270 |  |  |
| Number reconnected veins | 0.967(0.754-1.140) | 0.791 |  |  |
| PV RF duration | 1.000(1.000-1.001) | 0.742 |  |  |
| SVC duration | 1.002(0.999-1.004) | 0.139 |  |  |
|  |  |  |  |  |

BMI – body mass index; CAD – coronary artery disease; HT – hypertension; PLAX – parasternal long-axis (n=224); LVEF – left ventricular ejection fraction (n=262); LAVI – left atrial volume indexed (n=194).
